# Beyond killing: Can we find new ways to manage infection?

**DOI:** 10.1093/emph/eow012

**Published:** 2016-03-25

**Authors:** Pedro F. Vale, Luke McNally, Andrea Doeschl-Wilson, Kayla C. King, Roman Popat, Maria R. Domingo-Sananes, Judith E. Allen, Miguel P. Soares, Rolf Kümmerli

**Affiliations:** 1 Centre for Immunity, Infection and Evolution; 2 Institute of Evolutionary Biology, School of Biological Sciences, University of Edinburgh, Edinburgh EH9 3FL, UK; 3 The Roslin Institute and R(D)SVS, University of Edinburgh, Easter Bush EH25 9JT, UK; 4 Department of Zoology, University of Oxford, Oxford OX1 3PS, UK; 5 Institute for Genetics and Development of Rennes - CNRS UMR 6290, 2, Avenue Du Pr. Léon Bernard, Rennes 35043, France; 6 Institute of Immunology and Infection Research, School of Biological Sciences, University of Edinburgh, Edinburgh EH9 3FL, UK; 7 Instituto Gulbenkian De Ciência, Rua Da Quinta Grande, 6, Oeiras 2780-156, Portugal; 8 Department of Plant and Microbial Biology, University of Zürich, Switzerland

**Keywords:** infection, anti-virulence drugs, damage limitation, disease tolerance, microbiota, evolution

## Abstract

The antibiotic pipeline is running dry and infectious disease remains a major threat to public health. An efficient strategy to stay ahead of rapidly adapting pathogens should include approaches that replace, complement or enhance the effect of both current and novel antimicrobial compounds. In recent years, a number of innovative approaches to manage disease without the aid of traditional antibiotics and without eliminating the pathogens directly have emerged. These include disabling pathogen virulence-factors, increasing host tissue damage control or altering the microbiota to provide colonization resistance, immune resistance or disease tolerance against pathogens. We discuss the therapeutic potential of these approaches and examine their possible consequences for pathogen evolution. To guarantee a longer half-life of these alternatives to directly killing pathogens, and to gain a full understanding of their population-level consequences, we encourage future work to incorporate evolutionary perspectives into the development of these treatments.

## BEYOND KILLING

The emergence of widespread resistance to antibiotics is driving an intense search for alternative therapeutic approaches against bacterial pathogens [1, 2]. A major part of this effort focuses on discovering novel antimicrobial drugs [3, 4]. However, the evolution of drug resistance appears to be inevitable (despite some interesting exceptions such as the continued susceptibility of *Treponema pallidum* [5] and *Streptococcus pyogenes* [6] to penicillin), meaning that novel antimicrobial drugs will only offer at best a temporary solution [7, 8]. The discovery of novel antimicrobial drugs, while crucial, should advance alongside approaches that minimize the evolutionary potential of pathogens [9–11]. The high killing potential of current drugs is one of the strongest sources of selection exerted on pathogens, as evidenced by the rapid and consistent evolution of antibiotic resistance [8]. The reason why antimicrobial drugs lead to the evolution of drug resistance is simply natural selection, which leaves behind the pathogen strains most capable of surviving the deleterious effects of antimicrobial compounds. Novel therapeutic approaches should therefore aim to minimize the impact of this evolutionary response, and one way to do so is to control infections without killing pathogens directly. Here, we review three promising advances that move beyond direct killing to reduce disease severity, including: (i) the targeting of the effectors of pathogenicity rather than the pathogen itself; (ii) improving tissue damage control, thereby improving the host’s capacity to tolerate pathogens; and (iii) targeting the microbiome in order to build a natural line of defence against pathogens. For each approach, we introduce its mode of action, present key examples, and discuss putative selection pressures and evolutionary responses to treatment. Finally, we discuss the applicability of these approaches, and emphasize that it is imperative to investigate in more detail the longer-term evolutionary consequences of such treatments.

## DISARMING PATHOGEN VIRULENCE FACTORS

**Figure 1.**
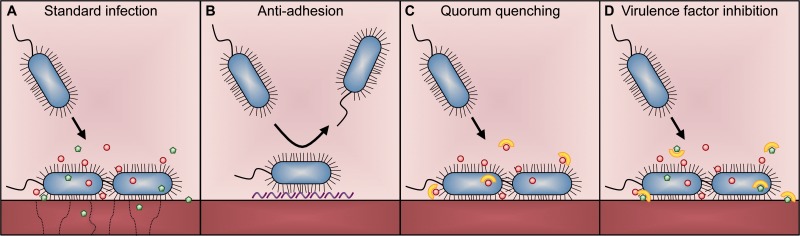
Examples of anti-virulence approaches. **(A)** In a classical infection, bacteria adhere to host tissue using their flagella and pilli. They then secrete quorum-sensing molecules (red dots) to communicate with nearby cells in order to coordinate the secretion of harmful virulence factors (green pentagons), such as toxins and tissue-degrading enzymes. **(B)** A potent anti-virulence approach is to prevent bacterial adhesion by the administration of hydrophilic compounds (purple layer) [15]. **(C)** Interference with bacterial communication, called quorum-quenching has been proposed as another efficient way to control bacterial infections. Numerous drugs (yellow half-circles) have been shown to either quench the bacterial signals outside the cell or to directly stall signal production within cells [26], **(D)** Approaches have also been developed to target the damaging virulence factors (e.g. siderophores, toxins) directly by either suppressing their synthesis or by inhibiting their actions once secreted [12]

One promising alternative to classic antibiotics is to focus on strategies reducing pathogen virulence, which we define in the broadest sense as the degree of pathology and overall disease symptoms experienced during infection. Pathogen virulence can be targeted at least at three levels (Fig. 1) [12–14], by interfering with: (i) pathogen adhesion, which is important for host invasion and colonization; (ii) quorum sensing, a cell-to-cell signalling system used by bacteria to coordinate the secretion of virulence factors; and (iii) expression and activity of virulence factors, which are usually secreted proteins or secondary metabolites that act directly or indirectly to cause tissue dysfunction and/or damage [9, 12]. Approaches belonging to these categories are called anti-virulence therapies, as they deprive essential virulence factors from infections without directly killing the pathogens themselves.

One example of such an anti-virulence drug approach was recently described in *Clostridium difficile* infections, where a synthetic compound called ebselen was found to be effective in inhibiting two major virulence-causing toxins (TcdA and TcdB) [16]. When tested in a mouse model, it was shown that ebselen reduced the disease severity of *C. difficile* infections without affecting the pathogen load [16]. Another example of pharmacological approaches targeting virulence factors is the use of phosphonosulphonates to treat *Staphyloccocus aureus* infection. These compounds inhibit the production of staphyloxanthin, a bacterial anti-oxidant pigment that normally protects *S. aureus* from reactive oxygen species and neutrophil-based killing. When staphyloxanthin is inhibited, *S. aureus* becomes vulnerable to innate immune resistance mechanisms, without interfering with commensal conspecifics [17]. Finally, a recent study successfully targeted the iron-scavenging capacity of *Pseudomonas aeruginosa*, through the administration of gallium, an iron-mimic, which binds to iron-scavenging siderophores produced by the pathogen. Gallium disables siderophores outside the cells, thereby preventing iron uptake and inducing iron-starvation [18]. Although these examples represent novel approaches to manage infection, it is also worth noting that some antibiotics, in addition to killing, can also exhibit anti-virulence effects. For example, both clindamycin and gentamycin can reduce the toxin production underlying toxic shock syndrome [19], and azithromycin can reduce expression of virulence genes in *P. aeruginosa* [20].

The above examples illustrate that effective anti-virulence treatments already exist. However, they also show that the initial idea of disarming pathogens without curbing their fitness might often not hold. For instance, phosphonosulphonates expose bacteria to host-mediated removal, and gallium induces iron starvation. Hence, the question about the evolutionary robustness of these therapies, which is required for their sustainable use in the long-term, needs closer examination. Theoretical work suggests that the evolution of resistance against anti-virulence is restricted if disarming a virulence factor has no fitness consequences for the pathogen [12]. Here, resistant variants might evolve but should not spread because they enjoy no fitness advantage compared with the susceptible wild type. Although it is difficult to imagine that bacteria express traits that have absolutely no effect on their growth and/or survival, there are at least a few examples (see the above-mentioned ebselen therapy, but also [21]) that seem to meet this criterion. Alternatively, it has been suggested that drug resistance against anti-virulence treatments should not easily spread if the therapy targets a virulence factor that is secreted and shared between pathogen individuals [22, 23]. In this scenario resistant mutants might restore production of the drug-inhibited virulence factor or produce a modified, more potent, version of it [18, 24]. However, these mutants should not spread because the freshly produced virulence factors are shared among cells and thereby benefit mutant and susceptible wild-type individuals alike. The above-mentioned gallium therapy falls within that category, because it targets secreted and publically shared siderophores. Evolutionary experiments indeed revealed no detectable signs of resistance against gallium, indicating that even drugs reducing pathogen growth can be evolutionarily robust, if they target shared virulence factors [18]. In addition to reducing the potential for resistance to spread, one could also seek to reduce the probability that resistant mutants arise in the first place. Although this point is typically considered in antimicrobial drug design [11], it has particular relevance for anti-virulence drugs that target secreted virulence factors outside the cell. The idea is simple: extra-cellular modes of drug actions should prevent common resistance mechanisms, such as limitation of drug entry, increased drug efflux or intra-cellular drug degradation, from operating [25]. Extra-cellular quenching of siderophores or quorum-sensing signals are approaches belonging to this category of treatments [18, 26].

These considerations suggest that anti-virulence therapies can be evolutionarily more robust than classic antibiotic treatments if the virulence factor in question: (i) has marginal fitness effects; (ii) is shared among individuals; (iii) and/or is disabled outside the cell. If this concept holds true we could not only use it as a guideline for future drug design, but also identify the approaches that are less likely to be evolutionarily robust. For instance, the above-described phosphonosulphonates therapy to treat *S. aureus* infection [6] does not belong to any of the three categories, as this therapy reduces pathogen fitness and targets a private and not a secreted shared virulence factor. Thus, it seems conceivable that resistance in the form of restoration of the defence against the host’s innate immune system could quite easily evolve. However, strong conclusions on the evolutionary robustness of anti-virulence therapies are currently not possible because we simply lack estimates of the strength selection imposed by different anti-virulence treatments on pathogens. This calls for studies that actually measure the selection pressures exerted by the different treatment schemes also taking into account any possible pleiotropic effects including undesirable changes in pathogen or host behaviour.

## TARGETING TISSUE DAMAGE CONTROL MECHANISMS TO ENHANCE HOST TOLERANCE OF INFECTIONS

In this section, we examine therapeutic approaches that strengthen the host’s ability to control and repair tissue damage. Although host mechanisms of pathogen elimination such as immune-mediated clearance are key to defence against pathogens, additional defence mechanisms which prevent, repair and limit the extent of tissue damage are also required to control infections [27]. Tissue damage control mechanisms are interesting from a therapeutic perspective because they enhance the capacity of an infected host to minimize disease severity, that is, to tolerate the pathogenic effects of infection [28, 29]. Disease tolerance may be defined as the host’s ability to maintain health when faced with increasing pathogen loads [28–30], and tissue damage control is one way to maintain health during infection [27]. Prevention or repair of tissue damage has been shown to confer disease tolerance of severe sepsis caused by polymicrobial infections [31], malaria caused by *Plasmodium* infection [32] and for co-infections by influenza virus and bacteria leading to pneumonia [33].

Recently, specific pharmacologic agents have been developed to specifically target tissue damage control mechanisms and to confer tolerance of infectious diseases. For example, a low dose regimen of anthracyclines has been shown to provide a protective effect during sepsis, preventing multi-organ dysfunction and damage even though the treatment does not reduce the bacterial load [34]. This example of tissue damage control leading to increased disease tolerance arises because anthracyclines induce a DNA-damage response that leads to the activation of autophagy-related pathways and reduction of systemic inflammation, the main cause of multi-organ dysfunction and damage associated with the pathogenesis of sepsis [34]. Targeting tissue damage control mechanisms therapeutically, as demonstrated as a proof of principle for anthracyclines could therefore be a promising alternative or addition to the widespread use of antibiotics if it can minimize the severity of infection while helping the host immune response to clear the infection. In many ways, treating the symptoms of infection rather than focusing on killing the root cause is not a new concept. Non-steroidal anti-inflammatory drugs are frequently used for the alleviation of symptoms for various infectious diseases. By treating the symptoms of infection without eliminating pathogens, these treatments are essentially tolerance-boosting therapies [9].

**Figure 2.**
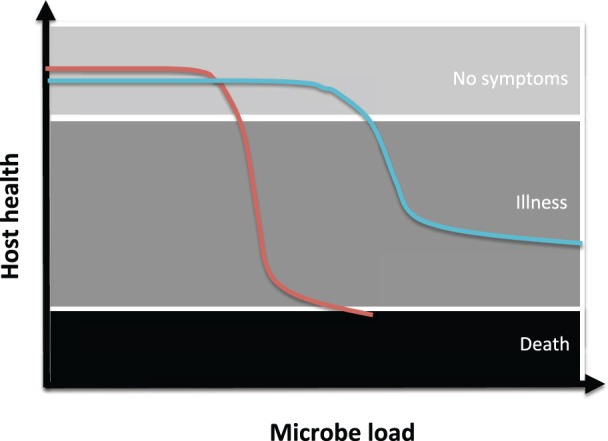
When comparing the ability of two different groups of hosts to limit damage during infection (e.g. a group with or without a damage control therapy), a common approach is to analyse how host health changes with increasing infection loads for each of the groups of interest. As pathogen loads increase during infection, hosts will lose health, going from a state of no symptoms to illness, and in extreme cases even death. In its simplest form, this relationship may be linear [30, 37], and host groups showing steep negative slopes for this reaction norm suffer a loss in health with increasing loads, while hosts with flat reaction norms are able to maintain health even as pathogen loads increase, and are therefore relatively tolerant. A potentially more realistic outcome is a non-linear relationship between host health and pathogen load. Hosts with more efficient damage prevention or repair mechanisms are able to maintain a higher level of health during infection (blue line) by affecting the sensitivity, slope or severity of the dose-response curve. The aim of therapies that promote tissue damage control is to flatten these relationships (by increasing the period before health plunges and/or lowering the slope)

One approach to uncover novel therapeutic targets for tissue damage control is to unravel the underlying causes for the enormous variation in disease tolerance that is often observed between species or even sub-species in their response to zoonotic pathogens. For example, bats, mice and humans are susceptible to infection by the Ebola virus, but these species have very different disease outcomes. It has been speculated that bats are especially capable of tolerating many zoonotic viruses through a combination of attenuated immunity—which reduces potential immunopathology—and the ability to minimize oxidative stress—an adaptation to metabolically costly activities like flight [35, 36]. The combined result is incomplete viral clearance and reduced immunopathology, which has been suggested as a plausible explanation for bats being such accomplished viral reservoirs, although concrete data to this effect is currently lacking. One way to compare groups of hosts that may differ in their ability to limit damage during infection is to obtain health read outs (e.g. survival, anaemia, immune markers) for increasing pathogen doses under controlled experimental conditions. These groups of hosts (e.g. different species as in the Ebola example, or human patients receiving damage limitation therapies) may differ in various parameters of this pathogen dose-host health response, including host vigour (the baseline level of health in the absence of infection), sensitivity to increases in pathogen load (the infection dose at which host suffer a severe decline in health) the rate at which host health decreases with increasing pathogen loads (the slope of the decline in health), or the severity of infection, which determines how sick a host can get during infection (Fig. 2). Variation in each of these parameters may reflect distinct underlying mechanisms that either promote greater prevention of damage during infection, or increase damage repair after the damage has been done [29]. If we were able to identify novel mechanisms of disease tolerance, we could then seek to develop therapies that enhance them with drugs that are likely to be more evolution-proof than conventional antibiotics.

Finally, it has been recognized that pathogen elimination mechanisms can work together with host mechanisms that promote tissue damage control and increase disease tolerance [30, 37, 38]. The idea is thus to develop therapeutics that strengthen the interaction between elimination and repair, especially in cases where pathogen elimination mechanisms fail, on their own, to reduce the pathogenesis of infectious diseases. For example, recent studies on mice infected with *Listeria monocytogenes* revealed that both the early immune-driven process leading to pathogen elimination and mechanisms that maintain host health at later stages of infection are important hallmarks for survival [39]. These insights were gained by tracing individual health trajectories during infection, constructed by plotting time-ordered, individual repeated measures of pathogen load against health throughout infection [40, 41]. This novel analytical approach can be used to monitor the interplay between pathogen elimination and host repair mechanisms and their combined effect on infection outcome, thus illustrating an individual’s infection path towards either recovery or death. The *Listeria* study described earlier [39] demonstrated that survivors and non-survivors of infection can assume divergent infection paths several days prior to death and that these paths are at least partly genetically determined [39]. This would suggest that one could predict the likely infection outcome based on characteristics of the infection paths at earlier stages of infection. Future studies should aim at determining the range of physiologically possible health trajectories associated with different types and outcomes of infections and at identifying the key regulatory mechanisms that are responsible for divergence in infection paths.

Alternatively to sequential dependence illustrated in [39], mechanisms that eliminate pathogens may overlap with tissue damage control mechanisms as illustrated by the immune response to parasitic worms. These parasites typically do not replicate in the host and, instead, represent a very different kind of threat; they typically injure or damage host tissues in order to enter, migrate or feed. Thus, infection by these parasites requires that any tissue damage be rapidly repaired and parasite numbers stay below a threshold that would compromise host fitness [42, 43]. This dual requirement has led to an immune response that relies on overlapping pathways to kill or expel the parasites, and to repair the damage they cause [44]. Thus the anti-worm effector responses have likely evolved directly out of wound healing pathways that confer tissue damage control [42, 43].

Although beneficial for the infected individual, the population-level consequences for pathogen evolution and spread of boosting host tolerance by improving tissue damage control have received relatively little attention [9]. The link between damage control, disease spread and pathogen evolution becomes intuitive when one recognizes that the ability of a pathogen to replicate and infect other individuals is constrained by how much damage it causes before its host dies (virulence) and how this level of virulence affect host evolutionary fitness [45]. Limiting tissue damage makes hosts healthier, but without eliminating pathogens directly potentially turns these hosts into disease reservoirs or silent spreaders [36, 46]. Evolutionary and epidemiological theory suggests that for infections where there is a strong link between virulence (infection-induced mortality) and pathogen fitness (the ability to replicate and spread to other hosts), therapies that limit tissue damage during infection can lead to the evolution of more virulent and also more prevalent infections [9, 47]. This link between virulence and pathogen fitness is particularly expected for obligate pathogens, where transmission between hosts is the main determinant of pathogen fitness. Conversely, this link is supposedly weaker for facultative pathogens, which can grow in non-disease contexts [48] and tolerance-boosting therapies are therefore expected to have fewer negative evolutionary consequences in this group of pathogens. Although these considerations suggest some caution, and highlight that it is necessary to balance the immediate benefits of alleviating virulence for individual patients with the potential longer-term costs for the population as a whole, the ubiquitous problem of antibiotic resistance makes it worth investigating if there are specific clinical scenarios where therapies promoting tissue damage control may be beneficial at both the individual and population levels.

## MANIPULATING COMMENSAL MICROBIOTA TO REDUCE INFECTION

A host’s health can be greatly impacted by its microbiome [49]. This realization has spurred momentum into studying the effects of microbes on animal and human biology, notably their role in altering host susceptibility to infection by different pathogens [49–53]. Bacteria, whether pathogenic or commensal, have evolved a battery of mechanisms to remove competitors and colonize their host, including the release of toxins and phage that directly kill competitors or provocation of host immune responses to which they are resistant, but their competitors are susceptible. Amongst pathogens prominent examples include the release of shiga toxin encoding phage in shigatoxinagenic *Escherichia coli* [54], production of the toxin pyocyanin by *P. aeruginosa* [55], recruitment of neutrophils into the paranasal spaces by *Haemophilus influenza* [56] and suicidal invasion of the gut tissue to provoke inflammation by *Salmonella enterica* serovar Typhimurium [57]. These examples show that there are strong competitive interactions between the commensal microbiome and pathogens, which opens the possibility for therapeutic interventions aiming at strengthening the microbiome and weakening the invasion potential of pathogens [58].

One prominent example of how protective bacteria can be used in therapeutic interventions is the treatment of *C. difficile* infections in humans. Briefly, *C. difficile* colitis occurs following perturbation of the host’s commensal microbiota, most commonly due to antibiotic treatment of unrelated infections [59]. A combination of evolved antibiotic resistance and intrinsic resistance factors such as spore formation, make that traditional antibiotic treatment often fails to eradicate *C. difficile* colitis [60]. An alternative therapy of ‘faecal transplant’ or bacteriotherapy, whereby the microbiome of the patient is repopulated using faecal material from healthy donors, has recently shown great promise, with high success rates in curing otherwise recurrent infections [61, 62]. Furthermore, the ecological basis of the success of this treatment has been mechanistically disentangled using a combination of sampling of human patients and experiments with a mouse model [63]. This study has shown that it is *C. difficle*’s cogener *Clostridium scindens* that protects against *C. difficile* infection by biosynthesis of secondary bile acids, which suppress *C. difficile* growth [63]. This work offers hope of using precise alterations to the human microbiota in order to protect against *C. difficile* infection.

Bacteriotherapy is not only a feasible defence against *C*. *difficile* infection; similar modifications of the gut microbiome have been suggested to treat a range of other infections. *Enterococcus faecalis* is a leading cause of hospital-acquired and often systemic infections with an increasing frequency of multi-drug resistant strains. However, many *E. faecalis* strains can also be a constituent of the normal healthy gut flora. Recent work in a mouse model of *E. faecalis* infection has shown that engineering strains of *E. faecalis* that express bacteriocin 21 encoded on conjugation-defective plasmids can clear infections of vancomycin resistant *E. faecalis* strains [64]. Strikingly, this treatment had no detectable effect on the composition of other species in the microbiota, with the treatment simply resulting in replacement of the virulent multi-drug resistant strain with a drug-susceptible commensal [64]. Similarly, it has been suggested that introducing commensal strains of *Staphylococcus epidermidis* could help reduce nasal carriage of drug resistant *S. aureus* via competitive exclusion [65]. As an alternative to genetic engineering, a novel approach would be to explore the degree to which natural variation in protective traits of single microbe species [58, 66] or whole microbiomes can be engineered through artificial selection. Experimental evolution approaches might prove very powerful in generating protective microbes and microbiomes with specific effects on disease defence.

These approaches are highly complementary to anti-virulence and tolerance increasing treatments, as introducing natural or engineered bacteria to a patient may be used to block pathogen virulence or promote tolerance. For instance, *Vibrio cholerae*’s virulence is negatively regulated by one of its quorum sensing molecules, cholera autoinducer 1 (CA-1). Introducing an engineered commensal *E. coli* strain that also produces CA-1 has been show to greatly reduce *V. cholerae* virulence in a mouse model, limiting binding of cholera toxin to the intestine by 80% and reducing *V. cholera* abundance by 69%, ultimately leading to an increase in host survival of 92% [67]. Specific components of the gut microbiota have also been shown to modulate disease tolerance [68]. For example, gut colonization by *E. coli* strain O21:H^+ ^confers a survival advantage against enteric and lung bacterial infections in mice without interfering with pathogen load, thus revealing that this *E. coli* strain induces host mechanisms that result in disease tolerance of bacterial infections [68]. The exact mechanism via which this occurs involves sensing of *E. coli* O21:H^+ ^triggering the induction of insulin-like Growth Factor-1, which acts systemically to alleviate muscle wasting [68]. It is this tissue damage control mechanism—in this case stimulated by a modification of the gut microbiota—that accounts for the survival advantage conferred by *E. coli* O21:H^+ ^against bacterial infections.

These examples highlight a great promise in effective microbiome-control therapies, yet understanding their evolutionary consequences is vital for assessing their suitability and sustainability as therapeutic approaches against infectious diseases. So far the consequences of alteration of the microbiome for pathogen evolution and epidemiology have received little attention [69], and we can therefore only speculate about possible evolutionary responses to microbiome therapy. Because these treatments aim to strengthen the opponents of the pathogen, they may create selection for increased expression of the pathogen’s arsenal of weapons used to clear commensals, potentially increasing their virulence. This adaptation of ‘fighting back’ is a likely outcome of pathogen evolution in cases where toxins are the direct causes of virulence in humans and are required to clear commensal competitors [69]. In addition to evolving to fight back against the strengthened microbiome, pathogens could evolve greater protection against its competitive effects. A recent experimental evolution study co-culturing *S. aureus* with the competitor *S. epidermidis* has shown that *S. aureus* can evolve resistance to the toxins used by *S. epidermidis* for competitive exclusion [65]. This study not only directly demonstrates that pathogens can evolve in response to the introduction of competitors, but highlights the utility of experimental evolution approaches to predict to potential responses to novel treatments. Additionally, one of the most common responses of bacteria to resist ecological competition is to form biofilms, which also greatly increase their resistance to antibiotics [70], and increases in biofilm formation could conceivably evolve in response to microbiome therapy. These considerations emphasize that, although bacteriotherapy is increasingly explored as a promising therapeutic approach against infections that are recalcitrant to traditional antibiotics, it is critical that their evolutionary consequences are elucidated to prevent unwanted repercussions arising from pathogen evolution.

## CONCLUSIONS

Pathogen evolution and the resulting resistance against treatments present a serious challenge to public health. Here, we propose three therapeutic approaches (disarming pathogens, boosting the host’s damage repair systems and strengthening the natural microbiome) that move away from direct pathogen killing to strategies that manage rather than eradicate infections. These approaches represent a fundamental conceptual shift in the way we think about infections, and could potentially be applied to both acute and chronic infections. Although all approaches look promising, a number of important questions remain to be addressed (Box 1). Because drug resistance evolution involves fundamental biological processes such as genetic variation and natural selection, managing these issues will only be successful if they are systematically addressed within an evolutionary ecology framework. First, a detailed mechanistic understanding of how virulence is mediated, and how hosts mount repair responses and interact with their microbiome is required. Only this knowledge will allow us to identify the most appropriate targets for evolutionarily robust and efficient therapies. In addition, interactions between the three approaches should be better understood. After all, virulence factors cause tissue damage and interfere with the microbiome, which opens the possibility for integrative therapies that simultaneously weaken the pathogen and strengthen the host. Second, a systematic theoretical framework is needed which examines the evolutionary robustness of the different approaches. It is important to realize that whatever therapy is used, it is likely to modify the within-host environment, and therefore inevitably imposes a different selection pressure on pathogens. Microbial adaptation to environmental changes, such as those imposed by therapy simply seems unavoidable, so it is vital that we investigate the potential epidemiological and longer-term evolutionary consequences of these new approaches to managing infections. Clearly, the urgent need for new strategies to fight infectious disease requires a close collaboration between scientists from molecular biology, evolutionary biology and medicine.

Box 1: Five outstanding questions

- What are the types and strengths of selection pressures that anti-virulence, tolerance and microbiome manipulation therapies impose on pathogens?
- Which virulence traits should be targeted to minimize selection on pathogens?
- What are the important components of host disease tolerance, and how can they be therapeutically enhanced to suppress disease in concert with host pathogen elimination mechanisms?
- How can the human microbiome be manipulated/strengthened to efficiently compete with pathogens?
- How do the three therapeutic approaches interact, and are there ways to synergistically combine them?
